# Working to advance scholarship is of vital importance to nursing in South Africa

**DOI:** 10.4102/hsag.v30i0.3269

**Published:** 2025-10-16

**Authors:** Gisela van Rensburg, Judy Bruce, Petra Brysiewicz

**Affiliations:** 1 Department of Health Studies, University of South Africa, Pretoria, South Africa; 2 School of Therapeutic Sciences, Faculty of Health Sciences, University of the Witwatersrand, Johannesburg, South Africa; 3 School of Nursing and Public Health, College of Health Sciences, University of KwaZulu-Natal, Durban, South Africa

Scholarship is central to advancing nursing knowledge and practice, primarily through research that both expands the evidence base and contributes to the profession. Scholarship in nursing means how professional knowledge is produced, collated and critiqued, communicated, and applied in practice, be it teaching practice, management practice, clinical practice, or research. Nursing scholarship aims at improving and advancing healthcare (in all its facets) and is the mainstay of the science and art of nursing (Academy of Nursing of South Africa [ANSA] 2023). Traditionally associated with research, scholarship encompasses the generation, organisation, evaluation, dissemination, and application of knowledge across various nursing domains, including teaching, management, clinical practice, and research. It serves as the foundation for improving healthcare, as well as strengthening and growing the profession by integrating scientific inquiry with practical expertise. The lack of scholarly engagement limits innovation and leadership in nursing (Boyer 2015; Pullen 2022). Historically, scholarship was seen as the domain of nurse educators and researchers in academic settings; however, clinical nurses and midwives, equipped with scientific and research skills, are vital in this process and contribute significantly to the development of nursing knowledge.

The ANSA, established in 2011, serves as a think tank dedicated to improving nursing education, practice, scholarship, and management. The ANSA has four strategic goals, one of which is the promotion of nursing scholarship in South Africa. Its position on nursing scholarship (ANSA 2023) is based on four key assumptions or truths:

- *Scholarship is a collective responsibility:* All members share the obligation to contribute to the integration of academia and practice.
- *Scholarship is setting-related, not setting-bound:* Knowledge generation and application occur in both academic and clinical settings.
- *Scholarship is fluid and dynamic:* It must evolve to accommodate diverse forms of engagement.
- *Scholarship strengthens nursing knowledge:* It builds a knowledge base that addresses community healthcare needs.

Developing a scholarly mindset fosters critical thinking, evidence-based practice, and professional growth, all of which are essential for enhancing patient care and the nursing profession. A nursing scholar is a well-educated, independent critical thinker who actively engages in intellectual pursuits, contributing and applying expertise to a specific subject or practice area (Boyer 2015; Pullen 2022). Scholars critique evidence-based materials, integrate research into teaching and practice, participate in professional and interprofessional forums, and lead initiatives that enhance nursing education and healthcare delivery. The scholarship of teaching, for instance, involves using innovative teaching methods, mentoring students, and fostering intellectual curiosity.

This is, however, not an easy task. Becoming a nursing scholar requires a great deal of deliberate effort and goal setting. We know that many barriers exist to scholarship of nursing in South Africa, namely under-resourced nursing schools with inadequate research infrastructure, a lack of emphasis on research within teaching roles (Bruce & Phetlhu 2024), a widening gap between academic and clinical practice, a lack of support for and mechanisms to recognise scholarship (Almaze, Emmamally & Brysiewicz 2023) and a culture of routineness and compliance that discourages critical thinking and innovation. In South Africa, many nurses receive their foundational education outside of university settings, and while efforts have been made to transition nursing qualifications towards a Bachelor’s degree, a significant gap remains in scholarship accessibility. Limited exposure to research, critical thinking, and evidence-based practice affects professional growth and the quality of care. The imbalance between nurses with advanced degrees and those in non-professional categories underscores the need for targeted strategies to bridge this gap. We also need to be cognisant of other global challenges facing nursing that play a part here. These include current nursing shortages and the ageing nurse population being experienced all over the world as well as high levels of nurse burnout and retention difficulties. The impact of climate change and the integration of technology and artificial intelligence are reshaping the nursing profession, presenting both challenges and opportunities for advancing nursing scholarship.

## So how do we then move forward?

Nurses are an essential component of any healthcare system. Nurses must be encouraged to question, reason, and seek new, alternative solutions, particularly in complex clinical settings where routine approaches may not suffice. The coronavirus disease 2019 (COVID-19) pandemic highlighted the vital role of nurses as frontline healthcare providers, but their contributions as leaders, educators, and policy influencers remain mute and in need of promotion and recognition. A strong culture of scholarship is essential for nurses to influence healthcare policies, lead quality improvement initiatives, and mentor future generations. Addressing these challenges requires accessible postgraduate programmes, mentorship opportunities, and institutional support for research. Professional development goals should include obtaining advanced education and certifications, and demonstrating expertise in a particular field through engaging in professional organisations, presenting research, and publishing in peer-reviewed journals.

In order to attempt to support such development, ANSA is committed to facilitating activities for nurses such as publication syndicates and interactive writing sessions; mentorship through wisdom circles; collaborative case studies linking practice and academia; interest groups on diversity, equity, and inclusion and by providing webinars, research cafes, and protected research discussions. By fostering such collaboration, ANSA is working to ensure that scholarship remains relevant and inclusive. It promotes the generation, utilisation, and dissemination of knowledge to strengthen the nursing profession and improve healthcare outcomes.

The evolution of nursing scholarship in South Africa requires a shift from an academic-centric model to one that includes clinical practitioners as active contributors. The ANSA is playing a pivotal role in bridging the gap between research and practice by fostering collaboration among educators, practitioners, and researchers. This is a call to action, a call to serve as an opportunity for nursing in South Africa to pause and reflect on how strengthening scholarship will enable South African nurses to drive innovation, improve patient care, and influence healthcare policy. Ultimately, scholarship matters because it empowers nurses to transform care delivery, elevate the profession, and address the nation’s unique healthcare challenges.
